# The Synergistic Effect of Zinc Ferrite Nanoparticles Uniformly Deposited on Silver Nanowires for the Biofilm Inhibition of *Candida albicans*

**DOI:** 10.3390/nano9101431

**Published:** 2019-10-10

**Authors:** Deepika Thakur, Saravanan Govindaraju, KyuSik Yun, Jin-Seo Noh

**Affiliations:** 1 Department of Nano-Physics, Gachon University, 1342 Seongnamdaero, Sujeong-gu, Seongnam-si, Gyeonggi-do 13120, Korea; thakurdeepz80@gmail.com; 2 Department of BioNano Technology, Gachon University, 1342 Seongnamdaero, Sujeong-gu, Seongnam-si, Gyeonggi-do 13120, Korea; biovijaysaran@gmail.com (S.G.); ykyusik@gachon.ac.kr (K.Y.)

**Keywords:** zinc ferrite nanoparticles, hybrid nanostructures, *Candida albicans*, biofilm inhibition, crystal violet assay

## Abstract

Near-monodisperse zinc ferrite nanoparticles (ZnFe_2_O_4_ NPs) are synthesized by a co-precipitation method and deposited on the surface of silver nanowires (AgNWs), employing a stepwise solution method. The resulting hybrid nanostructures (ZnFe_2_O_4_@AgNWs) show a thin and uniform layer of ZnFe_2_O_4_ NPs at an optimum weight ratio of 1:6 between the two component nanostructures. The hybrid nanostructures retain the high crystal quality and phase purity of their constituents. It is demonstrated that the ZnFe_2_O_4_@AgNWs hybrid nanostructures are effective at inhibiting the biofilm formation of *Candida albicans* cells. The biofilm inhibition activity of the hybrid nanostructures is estimated to be more than 50% at a low concentration of 100 µg/mL from both crystal violet assay and XTT assay, which are more than 8-fold higher than those of pure AgNWs and ZnFe_2_O_4_ NPs. This greatly enhanced biofilm inhibition activity is attributed to the ZnFe_2_O_4_ NPs-carrying membrane penetration by AgNWs and the subsequent interaction between *Candida* cells and ZnFe_2_O_4_ NPs. These results indicate that the ZnFe_2_O_4_@AgNWs hybrid nanostructures have great potential as a new type of novel antibiofilm agent.

## 1. Introduction

Typical candidiasis can proliferate into a severe fungal infection known as invasive candidiasis [1], which stimulates common oral thrush, vaginal and neonatal candidiasis, and bloodstream infections [2,3]. *Candida albicans* (hereafter abbreviated as *C. albicans*) is the prevailing species, responsible for almost 90% of invasive candidiasis [4], and usually resides as a normal commensal of the human oral, vaginal, and gut microflora [2]. Naturally, *C. albicans* grows in a symbiotic association within the human ecosystem that regulates the overgrowth and invasion of other tissues by communalistic colonization [5]. The regulation of overgrowth is an important factor to suppress parasitic interactions in order to develop into infections. Another known phenomenon that indicates the proliferation rate of *C. albicans* cells through intercellular signaling is quorum sensing [6]. This phenomenon is crucial for phenotypic changes in normal yeast cells. It is known that immunocompromised people, people with HIV or diabetes, and catheter-treated and long-term hospitalized patients are vulnerable to invasive candidiasis [7]. The normal *C. albicans* cells are easily transformed to hyphal morphogenesis (yeast­to­hyphae transition), promoting the formation of biofilm, a thick extracellular polymeric matrix (EPM) mainly consisting of proteins, lipids, and nucleic acid components [8]. The biofilm formation of *C. albicans* increases the resistance to commercial antifungal drugs due to the increased fungal activity in the film [9,10]. In the past two decades, there have been drastic morphological changes due to the yeast­to­hyphae transition of *Candida* species, and the associated risk of antifungal-drug resistance seems to be gradually increasing [11]. Epidemiological changes and ever-growing drug resistance can cause a high mortality rate of up to 60% for hospitalized patients with invasive candidiasis and related infections [12,13,14]. Therefore, it is necessary to regulate the proliferation from normal yeast infection to life-threatening invasive candidiasis, which is capable of infecting multiple organs including the central nervous system, brain, blood, heart, bones, etc.

The applications of nanoparticles (NPs) over conventional therapeutics and diagnostics are important since they can touch every area of human microbiological infection. However, there have been limited studies about *Candida* infection treatment with conventional NPs of silver (Ag), gold (Au), titanium (Ti), zinc (Zn), and their oxides [15,16,17]. For instance, Pierce et al. synthesized silver nanoparticles (AgNPs) to evaluate the *C. albicans* antifungal–biofilm activity at submicrogram concentrations, and showed good biocompatibility [18]. Another study on *C. albicans* cells using zinc oxide nanoparticles (ZnO NPs) turned out to be effective only for the inhibition of planktonic *C. albicans* growth [19]. New metal-resistant bacterial strains have, however, appeared with rapid industrialization and growing exposure to heavy metals, which could be a serious threat to fungal infection treatment in the future [20,21,22]. As an alternative, magnetic ferrite NPs such as cobalt (Co) ferrites, Ag-doped Co ferrites, and Zn ferrites have been evaluated for antifungal activity [23,24,25], although their study areas were limited [26]. For example, Jadhav et al. studied the anti-*Candida* adhesion activity of Zn ferrite (ZnFe_2_O_4_) NPs with an average size of ~40 nm [27]. Furthermore, the quorum sensing-mediated inhibition of biofilm using silver nanowires (AgNWs) was disclosed to be effective in both fungi and Gram-negative bacteria, but the high inhibitory concentration weakened their potential application as anti-*Candida* agents [28]. 

In this work, we synthesized Zn ferrite NPs-deposited AgNWs (ZnFe_2_O_4_@AgNWs) hybrid nanostructures to evaluate their biofilm inhibition activity for *C. albicans* at relatively low concentrations. To the best of our knowledge, these hybrid nanostructures have been explored for the first time for antifungal activity. AgNWs alone did not exhibit an antifungal biofilm effect, whereas the biofilm inhibition activity of AgNWs was greatly enhanced with the addition of ZnFe_2_O_4_ NPs, indicating that ZnFe_2_O_4_@AgNWs had synergistic effects on *C. albicans* as an effective antibiofilm agent.

## 2. Materials and Methods 

### 2.1. Chemicals and Reagents

All the necessary chemicals, including zinc nitrate hexahydrate (Zn(NO_3_)_2_·6H_2_O), iron nitrate nonahydrate (Fe(NO_3_)_2_·9H_2_O), silver nitrate (AgNO_3_), polyvinylpyrrolidone (PVP), ethylene glycol (EG, C_2_H_6_O_2_), copper (II) chloride dihydrate (CuCl_2_·2H_2_O), and sodium borohydride (NaBH_4_), were purchased from Sigma Aldrich (Yongin, South Korea). Other reagents like acetone, sodium hydroxide (NaOH), and ethyl alcohol (C_2_H_5_OH) were purchased from Daejung Chem. (Siheung, South Korea). All the chemicals and reagents were pure and used with no further treatment.

### 2.2. Synthesis of Hybrid Nanostructures

**Figure 1:**
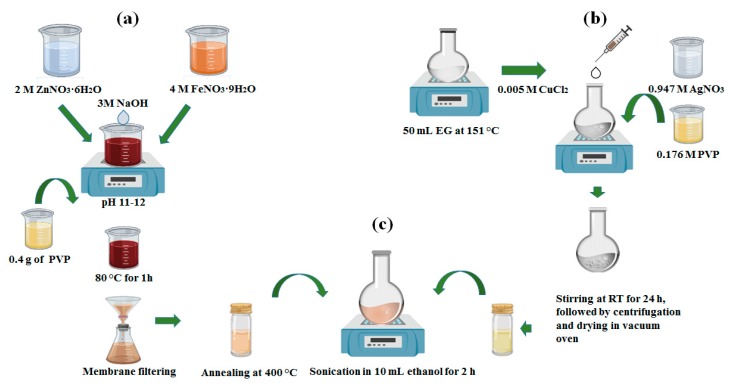
Synthesis procedures of (**a**) ZnFe_2_O_4_ NPs with PVP capping, (**b**) AgNWs, and (**c**) AgNWs combined with ZnFe_2_O_4_ NPs (ZnFe_2_O_4_@AgNWs hybrid nanostructures).

The synthesis of ZnFe_2_O_4_@AgNWs was performed in a stepwise manner, as schematically represented in Figure 1. A conventional coprecipitation method with PVP capping was followed for the synthesis of ZnFe_2_O_4_ NPs [29]. As presented in Figure 1a, FeNO_3_·9H_2_O (4 M) and ZnNO_3_·6H_2_O (2 M) were dissolved in 30 mL of deionized (DI) water, followed by the slow addition of NaOH (3 M). During the addition, the mixture solution was maintained at constant stirring until the pH reached 11–12. Then, a few drops of PVP solution (4 M) were added before heating at 80 °C for 1 h. Shortly, thick brown precipitates were cooled down to room temperature and collected by centrifugation after thorough washing with ethanol and DI water. Afterwards, the obtained product was dried in a heating oven at 105 °C and ground to fine particles with a mortar and pestle. Finally, the collected powder was annealed at 400 °C for 3 h and again collected by centrifugation. Before the final collection, the powder was filtrated using a 0.2-µm pore size membrane to achieve uniform size distribution by removing agglomerated particles. In parallel, AgNWs were synthesized using a polyol method, as depicted in Figure 1b. The detailed synthesis procedures were previously reported elsewhere [30]. Briefly, 50 mL of EG was stabilized by heating at 151.5 °C for 1 h, and AgNO_3_ solution (0.947 M) and PVP solution (0.176 M) were prepared using EG as a solvent. Next, 0.4 mL of CuCl_2_ solution (0.005 M) was added to the preheated EG. In 15 min, the AgNO_3_ and PVP solutions were added dropwise into the solution at a constant rate over 30 min, leading to the gradual color change from transparent to gray. Then, the solution was kept overnight with magnetic stirring and AgNWs were finally collected through repeated centrifugation and washing. The final step was the deposition of ZnFe_2_O_4_ NPs over the surface of AgNWs, as shown in Figure 1c. For that, collected powders of both ZnFe_2_O_4_ NPs and AgNWs were dispersed together in 10 mL of ethanol and sonicated for 2 h at room temperature. The ZnFe_2_O_4_ NPs and AgNWs were mixed at two different weight ratios (1:2 and 1:6). Finally, the ZnFe_2_O_4_@AgNWs hybrid nanostructures were collected by centrifugation after drying.

### 2.3. Characterization of Hybrid Nanostructures

As-synthesized powders of pure ZnFe_2_O_4_ NPs, pure AgNWs, and ZnFe_2_O_4_@AgNWs hybrid nanostructures were all characterized. For morphological examination, a field emission scanning electron microscope (FE-SEM, JEOL JSM-7500F, Tokyo, Japan) was used. The crystal structure and phase purity were analyzed by X-ray diffraction (XRD, Rigaku D/MAX 2200, Tokyo, Japan) with Cu *K*_α_ radiation. The oxidation state and the nature of bonding of constituents (Zn, Fe, O and Ag) in individual samples were examined by X-ray photoelectron spectroscopy (XPS, K-alpha Thermo Electron, Thermo Fisher Scientific, Waltham, MA, USA). The optical characteristics of the samples were checked using a UV-Vis spectrophotometer (UV-Vis Cary 50 Bio, San Diego, CA, USA). In addition, the photocatalytic activity of the hybrid nanostructures was evaluated under AM1.5 conditions, using a solar simulator (XEC-301S, Osaka, Japan) as a light source and methylene blue (MB) as a probe dye. For this test, 20 mg of ZnFe_2_O_4_@AgNWs were dispersed in 50 μL of MB solution (10 μM) and stirred well.

### 2.4. Strains, Media, and Culture Conditions 

The *Candida albicans* strain SC5314 was purchased from ATCC (Incheon Koram, South Korea) and stored at 80 °C until further experimental use. A loop of inoculum was streaked into yeast‒peptone‒dextrose agar (YPD, consisting of 2% yeast extract, 2% peptone, 1% dextrose, and 1.5% agar) on a Petri dish and incubated at 30 °C overnight. Then, the overnight grown culture was subcultured into a conical flask containing 30 mL of YPD broth media and incubated at 30 °C for 12–14 h in an orbital shaker at 180 rpm. After washing with phosphate-buffered saline (1× PBS), the cells were again resuspended in RPMI-1640 media (RPMI) with L-glutamine (Sigma Aldrich, Yongin, South Korea) and buffered in 0.165 mM 3-(N-morpholino) propanesulfonic acid (MOPS, Sigma Aldrich, Yongin, South Korea) as an essential supplement for *Candida* biofilm study [31]. The evolution of the *Candida* biofilms was evaluated by high-throughput screening, using a flat-bottomed 96-well microtiter plate (Corning, Inc., Wujiang, China) method [32]. Furthermore, the biofilm quantification was studied by a widely used crystal violet staining assay [33] and the colorimetric analysis using an 2,3-bis(2-methoxy-4-nitro-5-sulfo-phenyl)-2H-tetrazolium-5-carboxanilide (XTT) cell proliferation assay [34]. The detailed processes for the crystal violet assay and XTT cell proliferation assay are described in the Supplementary Materials, along with fluorescent dye staining assay. The starting culture of 1 × 10^6^ (CFU/mL) suspension was prepared using a hemocytometer cell-counting technique with 0.4% trypan blue dye to maintain cell density as a McFarland standard. For the biofilm treatment, the colloids of pure ZnFe_2_O_4_ NPs, pure AgNWs (0, 50, 100, 150, 200, 250, and 300 µg/mL), and ZnFe_2_O_4_@AgNWs (0, 10, 20, 40, 60, 80, and 100 µg/mL) were prepared in RPMI medium with varying the concentrations and stored at 4 °C. The comparative quantification analyses were performed using optical densities from control wells and treated wells at their inhibitory concentrations.

### 2.5. SEM Examination of C. albicans Biofilms

In order to prepare the samples for SEM examination, the *Candida* cells were treated with effective inhibitory concentrations of pure ZnFe_2_O_4_ NPs, pure AgNWs, and ZnFe_2_O_4_@AgNWs. A biofilm was developed at the bottom of the microtiter plate, using the method explained above. The biofilm formed at the bottom of microtiter plate was gently washed with PBS, and further sample preparation was conducted following a method reported previously [35]. In brief, the freshly grown biofilm was rinsed and fixed with 4% formaldehyde and 1% glutaraldehyde in PBS, followed by rinsing twice with a phosphate buffer (0.1 M). After a few minutes, 1% osmium tetroxide (OsO_4_) solution was added to the wells and kept there for 1 h. Then, the wells were progressively dried with solutions of increasing ethanol concentration (20%, 30%, 50%, 70%, 95%, and absolute ethanol, 10 min each). The SEM images were collected from both treated and untreated wells for the evaluation of biofilm morphologies. 

## 3. Results and Discussion

### 3.1. Morphologies

**Figure 2:**
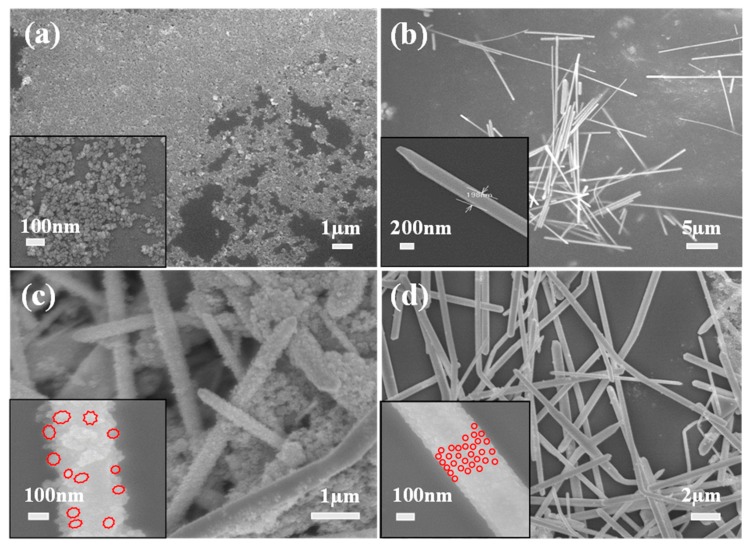
SEM images of (**a**) as-synthesized ZnFe_2_O_4_ NPs coated with PVP, (**b**) pure AgNWs, (**c**,**d**) ZnFe_2_O_4_@AgNWs hybrid nanostructures with different weight ratios: (**c**) ZnFe_2_O_4_ NPs:AgNWs = 1:2, (**d**) ZnFe_2_O_4_ NPs:AgNWs = 1:6. The red contours in the insets of (**c**,**d**) represent the general shapes of ZnFe_2_O_4_ NPs deposited on the surface of AgNWs.

The morphologies of all the nanostructures were characterized, and the representative SEM images are shown in Figure 2. The as-synthesized ZnFe_2_O_4_ NPs coated with PVP look monodisperse, but some degree of agglomeration is also observed due to the inter-particle magnetic interaction (Figure 2a). The average size of the nanoparticles is estimated at 33 nm. A SEM-EDX profile in Figure S1 clearly shows the presence of three elements (Z, Fe, O) in the nanoparticles. From Figure 2b, it is found that as-synthesized AgNWs are also uniform and well-faceted. There are no AgNPs found, indicating the dimensional purity of the nanostructures. The average diameter and length of AgNWs are estimated to be 200 nm and 15 µm, respectively. It is revealed that the morphologies of ZnFe_2_O_4_@AgNWs hybrid nanostructures depend on the weight ratio of ZnFe_2_O_4_ NPs to AgNWs, as shown in Figure 2c,d. At a lower ratio (ZnFe_2_O_4_ NPs:AgNWs = 1:2), ZnFe_2_O_4_ NPs completely cover the surface of AgNWs, and those NPs seem to be agglomerated even on the AgNW surface, leading to the rough surface (Figure 2c). The estimated particle size (~70 nm) is larger than that of as-synthesized ZnFe_2_O_4_ NPs. Moreover, many independent agglomerates of ZnFe_2_O_4_ NPs are also found. On the other hand, a thin and relatively smooth layer of ZnFe_2_O_4_ NPs is coated on the surface of AgNWs at a higher ratio (ZnFe_2_O_4_ NPs:AgNWs = 1:6) (Figure 2d). Consequently, the general shape of the hybrid nanostructures resembles that of pure AgNWs. From the inset of Figure 2d, no significant agglomeration of ZnFe_2_O_4_ NPs is observed on the surface. Based on this difference in morphologies, ZnFe_2_O_4_@AgNWs with a weight ratio of 1:6 were used for further examinations and biofilm inhibition studies.

### 3.2. Crystallographic, Chemical, and Optical Features of Hybrid Nanostructures

**Figure 3:**
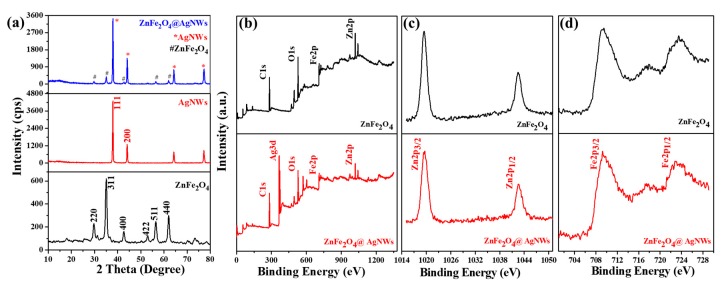
(**a**) XRD patterns of pure ZnFe_2_O_4_ NPs, AgNWs, and ZnFe_2_O_4_@AgNWs hybrid nanostructures. (**b–d**) XPS spectra of ZnFe_2_O_4_ NPs and ZnFe_2_O_4_@AgNWs hybrid nanostructures: (**b**) overall scans, (**c**) magnified spectra focused on Zn 2p levels, (**d**) magnified spectra focused on Fe 2p levels.

Figure 3a shows the XRD patterns of pure ZnFe_2_O_4_ NPs, pure AgNWs, and ZnFe_2_O_4_@AgNWs hybrid nanostructures. The pure ZnFe_2_O_4_ NPs exhibit characteristic diffraction peaks at 29.75°, 35.05°, 42.65°, 53.05°, 56.5°, and 62.05°, which are indexed to the (220), (311), (400), (422), (511), and (440) planes of ZnFe_2_O_4_ crystal with cubic spinel structure (JCPDS card No. 82-1049) [36]. The pure AgNWs show sharp diffraction peaks, particularly at 37.95° and 44.15°, which correspond to the (111) and (200) planes of FCC Ag crystal (JCPDS card No. 04-0783). Interestingly, the ZnFe_2_O_4_@AgNWs hybrid nanostructures reveal the major peaks of both AgNWs and ZnFe_2_O_4_ NPs at the same positions as those for the two components. For instance, the primary peaks corresponding to the Ag (111) and ZnFe_2_O_4_ (311) planes appear at 37.95° and 35.05° for both hybrid nanostructures and constituent nanostructures. Furthermore, the peak sharpness for ZnFe_2_O_4_@AgNWs hybrid nanostructures is not deteriorated at all, as compared to AgNWs and ZnFe_2_O_4_ NPs. These XRD results indicate that the hybrid nanostructures can be well synthesized by the stepwise solution method developed in this work, without any crystal damage or the formation of a mixed phase. 

XPS measurements were performed to further evaluate the chemical bonding states of the hybrid nanostructures. Figure 3b–d present the XPS spectra of pure ZnFe_2_O_4_ NPs and ZnFe_2_O_4_@AgNWs nanostructures. From the overall spectra shown in Figure 3b, it is found that both nanostructures include the constituents (Zn, Fe, O) of ZnFe_2_O_4_, but strong Ag peaks are observed only in the hybrid nanostructures (see Figure S2 for precise peak positions). Magnified Zn 2p and Fe 2p profiles for both samples are displayed in Figure 3c,d. Zn 2p_3/2_ and Zn 2p_1/2_ peaks are found at the same positions for pure ZnFe_2_O_4_ NPs and ZnFe_2_O_4_@AgNWs hybrid nanostructures (at 1019.6 and 1042.5 eV, respectively). Likewise, the peak positions of Fe 2p_3/2_ and Fe 2p_1/2_ for the two nanostructures are almost superposed at 709.4 and 723.4 eV, respectively. These results indicate that the bonding characteristics of ZnFe_2_O_4_ NPs remain unchanged during hybridization with AgNWs. Furthermore, the fact that the characteristic peaks of ZnO, which are supposed to appear at 1021.7 and 1044.9 eV, are not found, and the Zn 2p peaks are very sharp, indicates that Zn^2+^ ions at octahedral sites are surrounded by tetrahedrally positioned Fe ions [37,38,39]. In addition, of the other potential compounds, the Fe_2_O_3_ and Fe_3_O_4_ phases are not formed, the Fe 2p_3/2_ positions of which should be at 710.6 and 711.6 eV, respectively [40,41]. Thus, it might be stated that the ZnFe_2_O_4_ NPs are in pure spinel ferrite phase with negligible secondary compounds such as ZnO, Fe_2_O_3_, and Fe_3_O_4_.

Both ZnFe_2_O_4_ NPs and ZnFe_2_O_4_@AgNWs hybrid nanostructures appeared to optically respond over the broad spectrum range from visible light to UV, as seen from the UV-Vis absorption spectra in Figure S3. This is a desirable attribute for the photoactivated bio-applications. To evaluate the photoactivity of the hybrid nanostructures, the time-dependent degradation of methylene blue (MB) was tested. As presented in Figure S4, the MB solution is greatly discolored after 80 min of light illumination. The photoactivated decomposition of MB is estimated at 98.5% for the 80-min-long illumination, which was calculated from the relative absorbance intensities at a characteristic peak (665 nm) before and after illumination. These results support the strong photoactivity of the hybrid nanostructures, although the decomposition time is longer than the previous reports [42], presumably due to the limited fraction (1/7 of the total weight) of ZnFe_2_O_4_ NPs in the nanostructures. 

### 3.3. Quantification of Biofilms by Crystal Violet Assay

**Figure 4:**
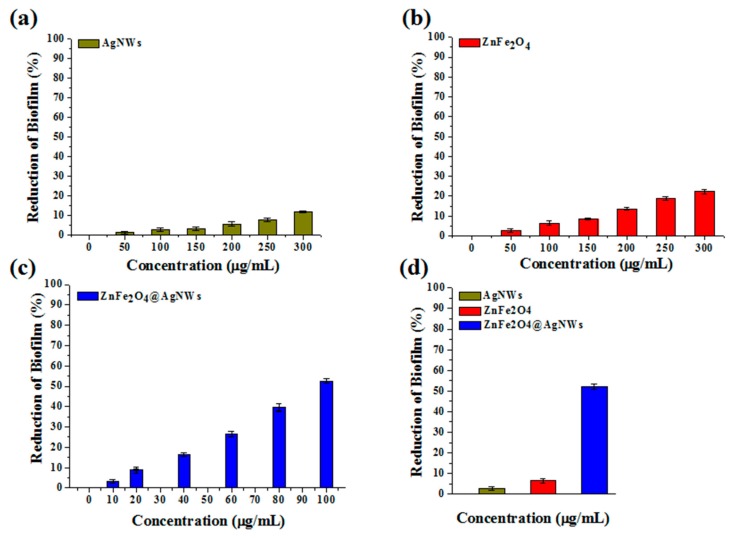
Reduction of biofilm versus nanostructure concentration bar charts obtained from crystal violet staining assays: (**a**) AgNWs, (**b**) ZnFe_2_O_4_ NPs, (**c**) ZnFe_2_O_4_@AgNWs hybrid nanostructures, (**d**) comparative reduction of biofilm at a specific concentration of 100 µg/mL. The reduction of biofilm was calculated from the optical density at the wavelength of 595 nm.

Quantification of biofilms was first made using crystal violet staining assay. Figure 4 shows the reduction of biofilm of control and treated wells depending on the concentration of individual nanostructures. The reduction charts were converted from the optical density charts, which are presented in Figure S5. For all the nanostructures, it is disclosed that the reduction of biofilm consistently increases as the nanostructure concentration increases. However, the degree of biofilm reduction, which represents the biofilm inhibition activity, differs with the type of nanostructures employed. Here, the biofilm inhibition activity (*I*) is calculated as Equation (1):

*I*(%) = (OD_0_ − OD_n_)/OD_0_ × 100 (1)

where OD_0_ and OD_n_ are optical densities from control wells and nanostructure-treated wells. The crystal violet staining assay treated with pure AgNWs exhibits only 11% of inhibition activity at a 300 µg/mL concentration (Figure 4a). Even this inhibition seems better than that of a previous report [28]. The inhibition activity of pure ZnFe_2_O_4_ NPs is estimated at 22.3% of the same concentration (Figure 4b). The assay treated with ZnFe_2_O_4_@AgNWs hybrid nanostructures reveals 52.2% of biofilm inhibition at a concentration of 100 µg/mL (Figure 4c), which is just one-third of the maximum concentration for the other two nanostructures. To better represent the relative inhibition activities of the three nanostructures, the optical densities of nanostructure-treated wells are compared at the same concentration of 100 µg/mL in Figure 4d. From the comparative data, the antibiofilm activities toward *C. albicans* are calculated to be 2.8%, 6.4%, and 52.2% for pure AgNWs, pure ZnFe_2_O_4_ NPs, and ZnFe_2_O_4_@AgNWs hybrid nanostructures, respectively. The enhanced biofilm inhibition activity of ZnFe_2_O_4_@AgNWs hybrid nanostructures is attributed to the synergistic effect of AgNWs and ZnFe_2_O_4_ NPs. Even though ZnFe_2_O_4_ NPs may play a greater role than AgNWs do, increasing the weight fraction of ZnFe_2_O_4_ NPs did not lead to improved biofilm inhibition activity, as confirmed in Figure S6.

### 3.4. Quantification of Biofilms by XTT Assay 

**Figure 5:**
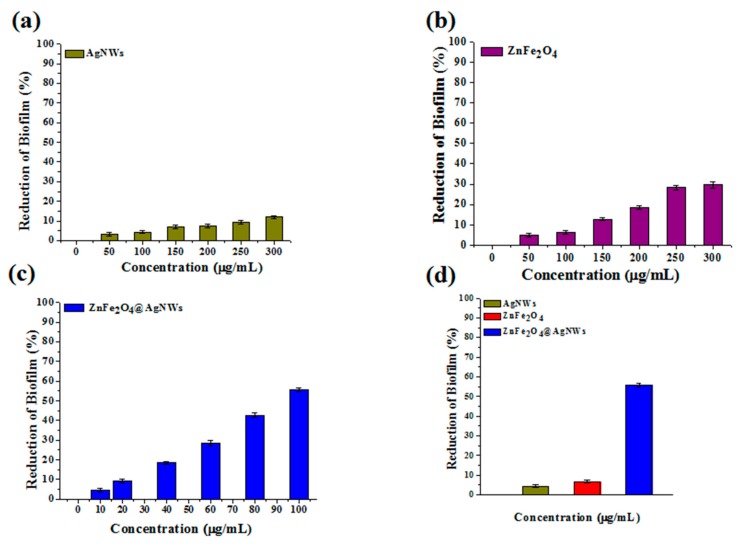
Reduction of biofilm versus nanostructure concentration bar charts obtained from XTT cell proliferation assays: (**a**) AgNWs, (**b**) ZnFe_2_O_4_ NPs, (**c**) ZnFe_2_O_4_@AgNWs hybrid nanostructures, (**d**) comparative reduction of biofilm at a specific concentration of 100 µg/mL. The reduction of biofilm was converted from the optical density at a wavelength of 490 nm.

The XTT cell proliferation assay was used to quantify the metabolic activity of *C. albicans* cells [43]. Like in the crystal violet staining assay method, varying concentrations of nanostructures were tested separately, and the results are presented in Figure 5 (also see Figure S7 for the corresponding optical density charts). Similar to the previous method, the reduction of biofilm gradually increases with an increase in the nanostructure concentration, indicating that all the nanostructures have the more or less biofilm inhibition activity. From Figure 5a,b, the biofilm inhibition activities of pure AgNWs and pure ZnFe_2_O_4_ NPs are estimated at 12% and 29.7%, respectively, at a concentration of 300 µg/mL. In contrast, the ZnFe_2_O_4_@AgNWs hybrid nanostructures show a great enhancement in the inhibition activity even at a reduced concentration: 55.7% at 100 µg/mL (Figure 5c). This inhibition activity is in very close agreement with that estimated from the crystal violet staining assay. Figure 5d clearly demonstrates the relative effects of individual nanostructures on the biofilm inhibition at a specific concentration of 100 µg/mL. The biofilm inhibition activities of pure AgNWs, pure ZnFe_2_O_4_ NPs, and ZnFe_2_O_4_@AgNWs hybrid nanostructures are calculated to be 4.3%, 6.2%, and 55.7%, respectively.

### 3.5. Nanostructure-Dependent Biofilm Inhibition

**Figure 6:**
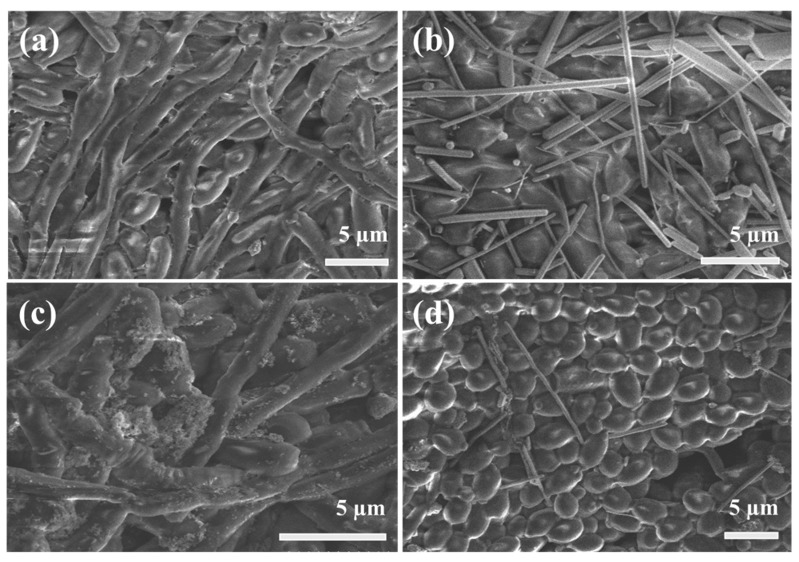
SEM images of (**a**) control *Candida* biofilm and biofilms treated with (**b**) 300 µg/mL of AgNWs, (**c**) 300 µg/mL of ZnFe_2_O_4_ NPs, and (**d**) 100 µg/mL of ZnFe_2_O_4_@AgNWs hybrid nanostructures. The biofilms were formed for 24 h.

The development of biofilms in a control sample and nanostructure-treated samples was examined by SEM. Figure 6 displays SEM images of *Candida* biofilms after 24 h of biofilm development. As can be seen in Figure 6a, the fully matured *Candida* biofilm with a long, thick, and complex hyphae structure is observed in the control sample without any treatment. There is no clear appearance of budding cells due to the biofilm covering almost all the surface. When treated with a 300 µg/mL of AgNWs, the nanowires are randomly distributed over the surface and partially penetrate inside the biofilm matrix (Figure 6b). Unfortunately, the pure AgNWs are not so effective for biofilm inhibition, even though some penetrating AgNWs can participate in the early cell death or adhesion competition to the surface of the microtiter plate. Likewise, an equal concentration of pure ZnFe_2_O_4_ NPs was not capable of inhibiting the biofilm growth. From Figure 6c, it is apparent that the ZnFe_2_O_4_ NPs are not able to penetrate the biofilm matrix as they stick to the upper surface due to the severe agglomeration. On the contrary, the growth of budding cells with clear round cell boundaries is clearly found in the sample treated with ZnFe_2_O_4_@AgNWs hybrid nanostructures, while the hyphal growth is effectively suppressed, demonstrating the synergistic effect of the hybrid nanostructures (Figure 6d). Additional SEM measurement and EDX analysis were performed for another sample treated in the same way, but after making a scratch. It is noted from Figure S8 that an AgNW is located below the surface, and all elements of ZnFe_2_O_4_@AgNW are detected near the AgNW, although the dominating element is C, which is the main component of polymeric matrix.

### 3.6. Biofilm Inhibition Mechanism 

**Figure 7:**
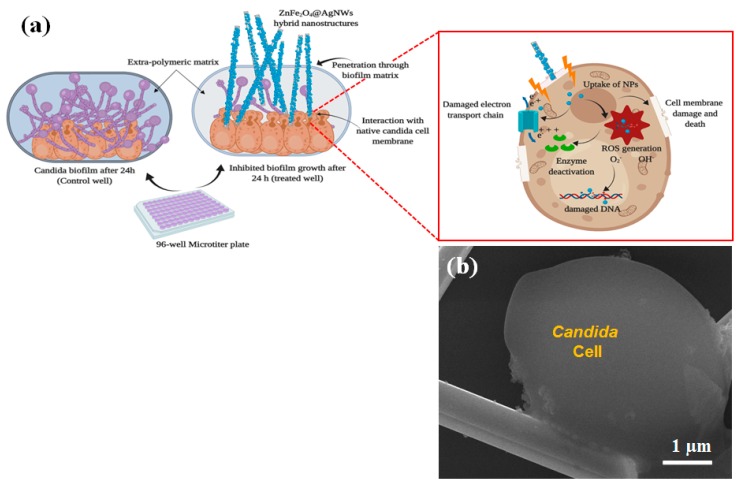
(**a**) Schematic illustration of the biofilm inhibition mechanism. (**b**) High-resolution SEM image of *Candida* biofilm treated with ZnFe_2_O_4_@AgNWs hybrid nanostructures (100 µg/mL) after 24 h of biofilm formation.

Figure 7 shows a plausible biofilm inhibition mechanism and a high magnification SEM image of the sample treated with a 100 µg/mL of ZnFe_2_O_4_@AgNWs hybrid nanostructures. As depicted in Figure 7a, the hybrid nanostructures may penetrate through the periphery of the *Candida* biofilm, reach the native *Candida* cells, and cause damage to the cells. Through this process, the hybrid nanostructures cause physical deformation and rupture in both the biofilm matrix and the cell membrane, and may interrupt the cell membrane’s function [18,44,45]. At the same time, the ZnFe_2_O_4_ NPs deposited on the surface of AgNWs play a significant role in the cell-damaging process by generating reactive oxygen species (ROS), which attack the cells. The ZnFe_2_O_4_ NPs are known to have unique properties such as high surface energy, good anti-adhesion, and easy binding with cell membrane components [46]. The ZnFe_2_O_4_ NPs, which may be bound to or released from the AgNW surface, exert oxidative stress on cells’ organelles, leading to the generation of ROS. Subsequently, the generated ROS damage the biological metabolism of the cells by enzyme deactivation and DNA damage, ultimately causing cell death [47]. As a consequence, the *Candida* cells become rougher and swollen, as confirmed in Figure 7b. The ZnFe_2_O_4_@AgNWs hybrid nanostructures can transport the ZnFe_2_O_4_ NPs inside the *Candida* biofilm without any agglomeration. Consequently, they show overwhelming biofilm inhibition activity at a relatively lower concentration, proving their effectiveness as a new antibiofilm agent.

### 3.7. Visualization of Candida Biofilms by Fluorescent Microscopy

**Figure 8:**
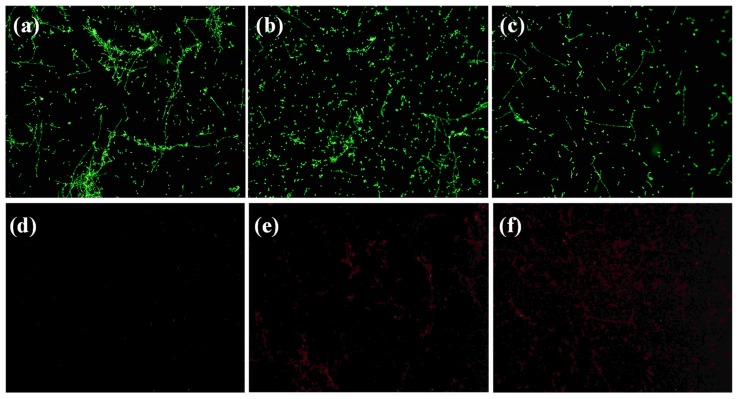
Fluorescent microscope images of *Candida albicans* biofilms, showing (**a–c**) live cell distributions and (**d–f**) dead cell distributions. (**a**,**d**) Control biofilms, (**b**,**e**) biofilms treated with ZnFe_2_O_4_@AgNWs hybrid nanostructures for 12 h, (**c**,**f**) biofilms treated with ZnFe_2_O_4_@AgNWs hybrid nanostructures for 24 h. Measurements were made using a 10× objective lens.

We stained a control biofilm and other biofilms treated with ZnFe_2_O_4_@AgNWs hybrid nanostructures (100 µg/mL) for different times, following the protocol described in the yeast visibility kit (Molecular Probes, Invitrogen, Thermo Fisher Scientific, Waltham, MA, USA). The biofilm images obtained by a fluorescent microscope are shown in Figure 8. For this imaging, two types of dyes (SYTO9 and propidium iodide) were used, which behave differently. SYT09 can penetrate cell membranes in both healthy and dead cells/hyphae, while propidium iodide is unable to pass through the cell membrane and binds to dead or damaged cells/hyphae. As a consequence, healthy cells appear green, whereas dead or damaged cells show red fluorescence. As can be seen in Figure 8a, the control biofilm shows strong, streaked green fluorescence, indicating active hyphal growth. Less hyphal growth and more *C. albicans* cells without hyphal development are observed for the 12-h-treated biofilm (Figure 8b). Further inhibition of hyphal growth and a lower density of *C. albicans* cells resulted in the biofilm treated for 24 h (Figure 8c). The distributions of dead or damaged cells (red fluorescence) for the same samples have the opposite trend to these healthy cell distributions (see Figure 8d–f). These results agree well with the observations from crystal violet assay, XTT assay, and SEM studies.

## 4. Conclusions

A stepwise solution method was utilized to synthesize new hybrid nanostructures composed of ZnFe_2_O_4_ NPs and AgNWs. The ZnFe_2_O_4_@AgNWs hybrid nanostructures retained the crystal structures and phase purities of their component nanostructures. The hybrid nanostructures were tested for their biofilm inhibition activity on *C. albicans* cells, in comparison with pure ZnFe_2_O_4_ NPs and pure AgNWs. Using biofilm quantification assays such as crystal violet staining assay and XTT cell proliferation assay, it was demonstrated that the ZnFe_2_O_4_@AgNWs hybrid nanostructures were indeed effective at suppressing the biofilm development of *C. albicans* cells. The biofilm inhibition activity was estimated to be more than 50%, even at a low concentration of 100 µg/mL. From the biofilm visualizations using SEM and fluorescent microscopy, the biofilm inhibition activity of the hybrid nanostructures was further confirmed. These were made possible by the synergistic effect of ZnFe_2_O_4_ NPs and AgNWs. AgNWs could penetrate the biofilm matrix and transport ZnFe_2_O_4_ NPs inside the biofilm, where the ZnFe_2_O_4_ NPs caused damage to native *Candida* cells by the generation of ROS. There was no agglomeration of ZnFe_2_O_4_ NPs found during cell treatment or penetration through the biofilm. From these results, the ZnFe_2_O_4_@AgNWs hybrid nanostructures could be suggested as a new and novel antibiofilm agent.

## Supplementary Materials

The following are available online at https://www.mdpi.com/2079-4991/9/10/1431/s1, Figure S1: SEM-EDX spectrum of pure ZnFe_2_O_4_ NPs, Figure S2: Magnified XPS spectrum of ZnFe_2_O_4_@AgNWs hybrid nanostructures, Figure S3: UV-Vis absorption spectra of (**a**) pure ZnFe_2_O_4_ NPs and (**b**) ZnFe_2_O_4_@AgNWs hybrid nanostructures, Figure S4: Photocatalytic degradation of methylene blue (MB) using ZnFe_2_O_4_@AgNWs hybrid structures, Figure S5: Optical density versus nanostructure concentration bar charts obtained from crystal violet staining assays, Figure S6: Optical density versus concentration bar chart for ZnFe_2_O_4_@AgNWs hybrid nanostructures with the weight ratio of 1:2, Figure S7: Optical density versus nanostructure concentration bar charts obtained from XTT cell proliferation assays, Figure S8: SEM-EDX spectrum of a biofilm sample treated with ZnFe_2_O_4_@AgNWs hybrid nanostructures.
