# Supplementary material for: The Synergistic Effect of Zinc Ferrite Nanoparticles Uniformly Deposited on Silver Nanowires for the Biofilm Inhibition of Candida albicans
Source: Nanomaterials (Basel). 2019 Oct 10;9(10):1431. doi: 10.3390/nano9101431 (PMC6835689; doi:10.3390/nano9101431)
Supplement: Supplementary file 1 [file nanomaterials-09-01431-s001.pdf]

Supporting Information

The Synergistic Effect of Zinc Ferrite Nanoparticles Uniformly Deposited on Silver Nanowires for the Biofilm Inhibition of *Candida Albicans*

Deepika Thakur [1], Saravanan Govindaraju [2], KyuSik Yun [2] and Jin-Seo Noh [1,*]

[1] Department of Nano-Physics, Gachon University, 1342 Seongnamdaero, Sujeong-gu, Seongnam-si, Gyeonggi-do 13120, Korea; thakurdeepz80@gmail.com (D.T.)

[2] Department of BioNano Technology, Gachon University, 1342 Seongnamdaero, Sujeong-gu, Seongnam-si, Gyeonggi-do 13120, Korea; biovijaysaran@gmail.com (S.G.); ykyusik@gachon.ac.kr (K.Y.)

* Correspondence: jinseonoh@gachon.ac.kr; Tel.: +82-317505611

1. Crystal violet staining assay for biofilm quantification

Crystal violet staining assay is more convenient assay for biofilm quantification.[1] For that, 100 µl of 1 × 10^6 candida suspension was incorporated into a 96-well microtiter plate. Then, 100 µl of as-prepared media with pure $ZnFe_2O_4$ NPs, pure AgNWs, and $ZnFe_2O_4$@AgNWs were incorporated into the wells and incubated at 37 °C for 24 h. Next, freshly grown biofilms were gently washed with PBS buffer 2-3 times and fixed with absolute ethanol. Subsequently, 100µl of freshly prepared 0.1% crystal violet solution was added into the wells and incubated at room temperature for 30 min. After removing the excess stain with DI water, 200 µl of acetic acid (33%) was added to solubilize the insoluble crystal violet bound to the cellular matrix. The optical density was read at a wavelength of 595 nm, and the effective inhibitory concentration was calculated using Origin8.5 Pro.

2. XTT cell proliferation assay for biofilm quantification

The XTT cell proliferation assay was utilized as another approach to study biofilm quantification. In order to perform the quantification of biofilm, 100 µl of initial cell suspension was harvested for biofilm formation with freshly prepared treatment agent, and 100 µl of media containing pure $ZnFe_2O_4$ NPs, pure AgNWs, and $ZnFe_2O_4$@AgNWs were added and followed by incubation at 37 °C for 24 h. Likewise, the planktonic cells were removed by gentle washing with sterilized PBS buffer 2-3 times, and then the test samples were resuspended in 100 µl of fresh RPMI media and 50 µl of XTT solution. The microtiter plate was further incubated at 37 °C for 3 h in a dark environment. The tetrazolium salt reduction by dehydrogenase enzyme of viable cells are responsible for the appearance

of dark red-colored water-soluble formazan solution.[2] Finally, the solution was transferred to a new microtiter plate for optical density measurement at a wavelength of 490 nm.

3. Fluorescent dye staining assay for biofilm visualization

We utilized the Live/Dead FungaLight yeast viability kit (Moleular Probes, Invitrogen) for visualization of live biofilm content after treatment with minimum inhibitory concentration (100 μg/ml) of $ZnFe_2O_4$@AgNWs hybrid nanostructures. The samples were prepared as described in the protocol for yeast viability kit.[3] The live (green) and dead (red) biofilms appear visible with respective fluorescence due to the cell membrane penetrating ability of SYTO9 dye and propidium iodide.

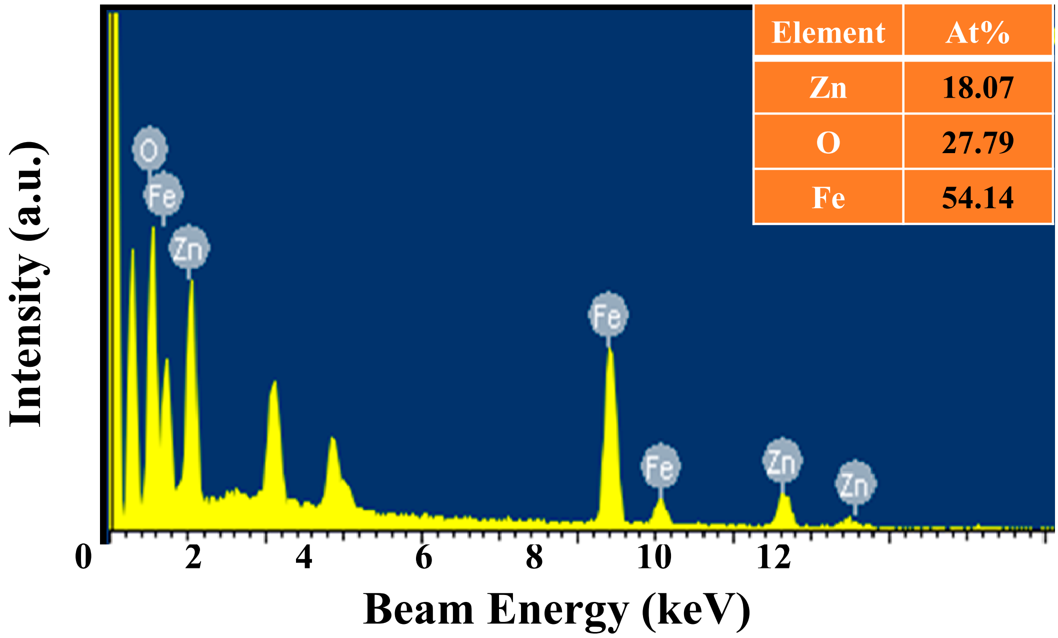

Element	At%
Zn	18.07
O	27.79
Fe	54.14

Figure S1. SEM-EDX spectrum of pure $ZnFe_2O_4$ NPs.

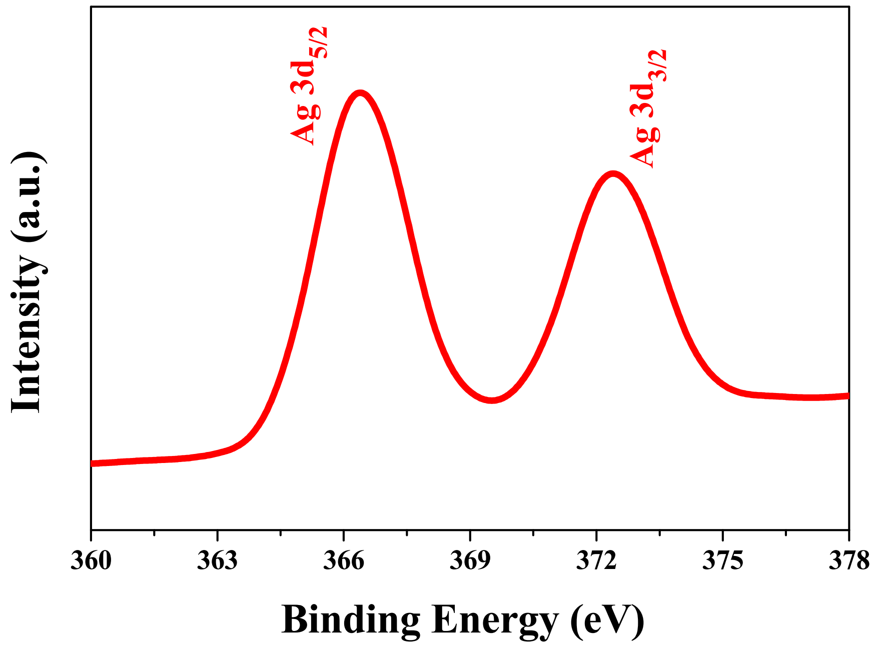

Figure S2. Magnified XPS spectrum of $ZnFe_2O_4$@AgNWs hybrid nanostructures focused on Ag 3d levels.

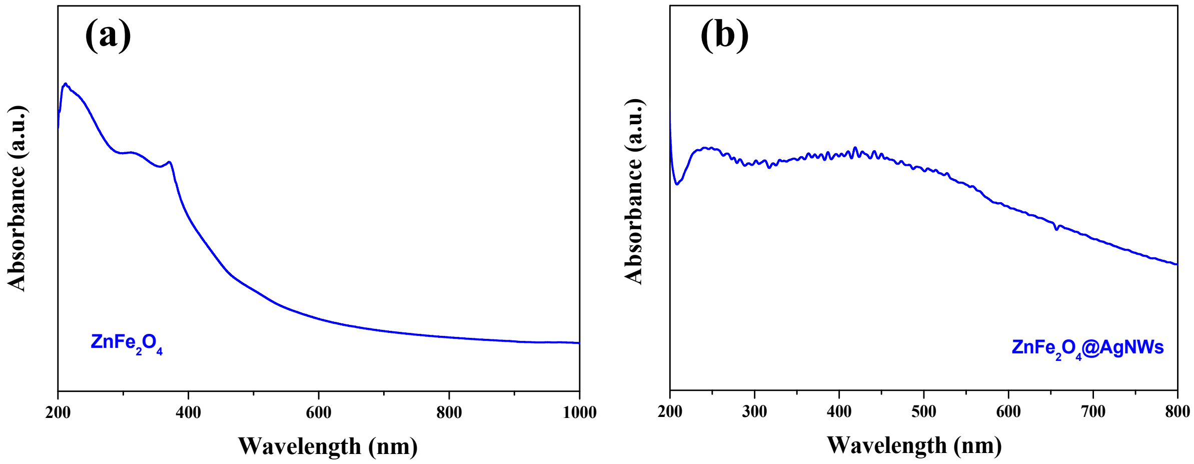

Figure S3. UV-Vis absorption spectra of (a) pure $ZnFe_2O_4$ NPs and (b) $ZnFe_2O_4$@AgNWs hybrid nanostructures.

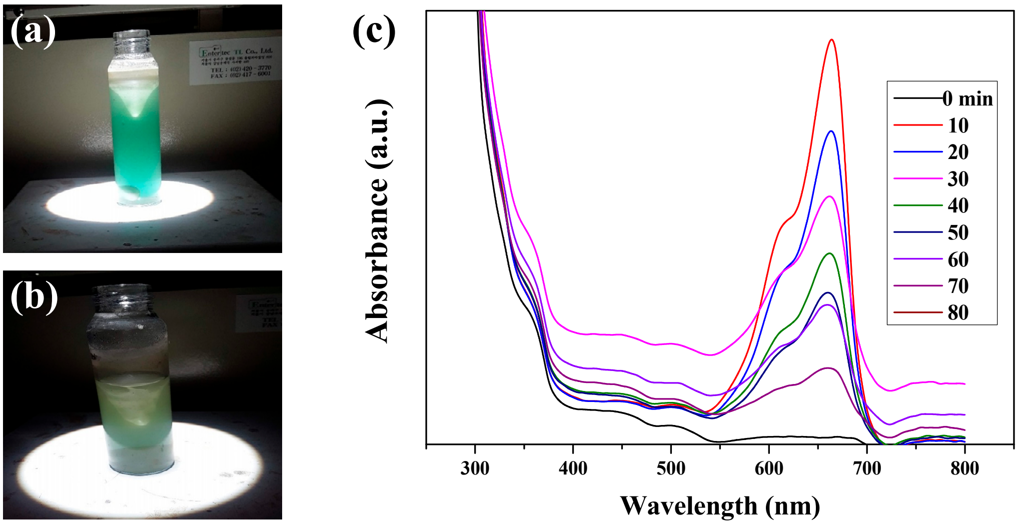

Figure S4. Photocatalytic degradation of methylene blue (MB) using $ZnFe_2O_4$@AgNWs hybrid structures. Photo images of the MB solution (a) before light irradiation and (b) after 80 min of light irradiation. (c) The change in absorption peak intensity depending on the irradiation time.

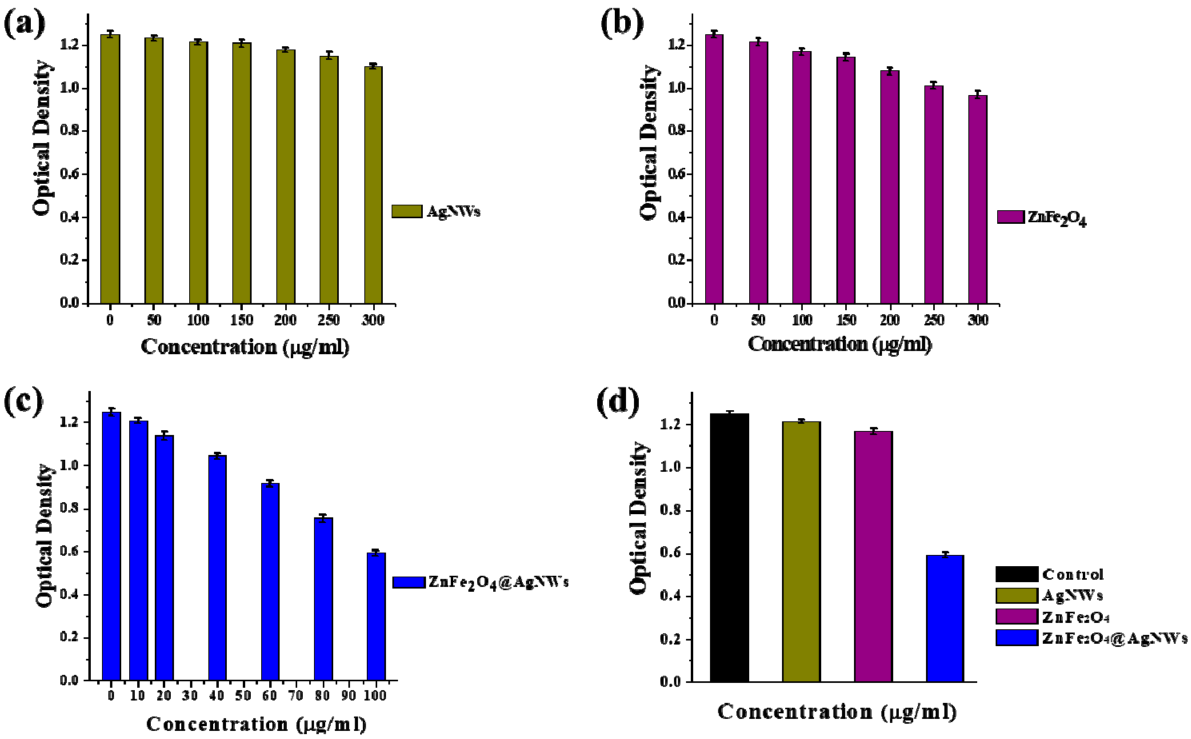

Figure S5. Optical density versus nanostructure concentration bar charts obtained from crystal violet staining assays: (a) AgNWs, (b) $ZnFe_2O_4$ NPs, (c) $ZnFe_2O_4$@AgNWs hybrid nanostructures, (d) comparative optical densities at a specific concentration of 100 μg/ml. The optical densities were converted from the light absorbance at the wavelength of 595 nm.

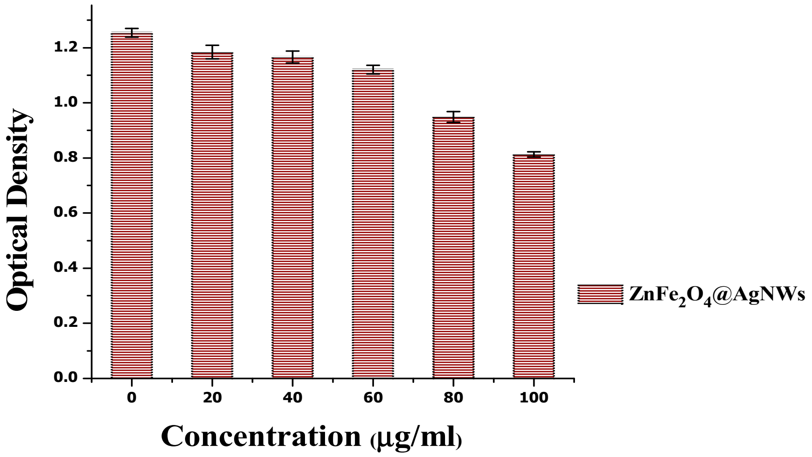

Figure S6. Optical density versus concentration bar chart obtained from a crystal violet staining assay. Here, $ZnFe_2O_4$@AgNWs hybrid nanostructures with the weight ratio of 1:2 were used for the test. At a concentration of 100 µg/ml, the biofilm inhibition activity is estimated at 34.7%, which is much lower than the value (52.2%) for the weight ratio of 1:6.

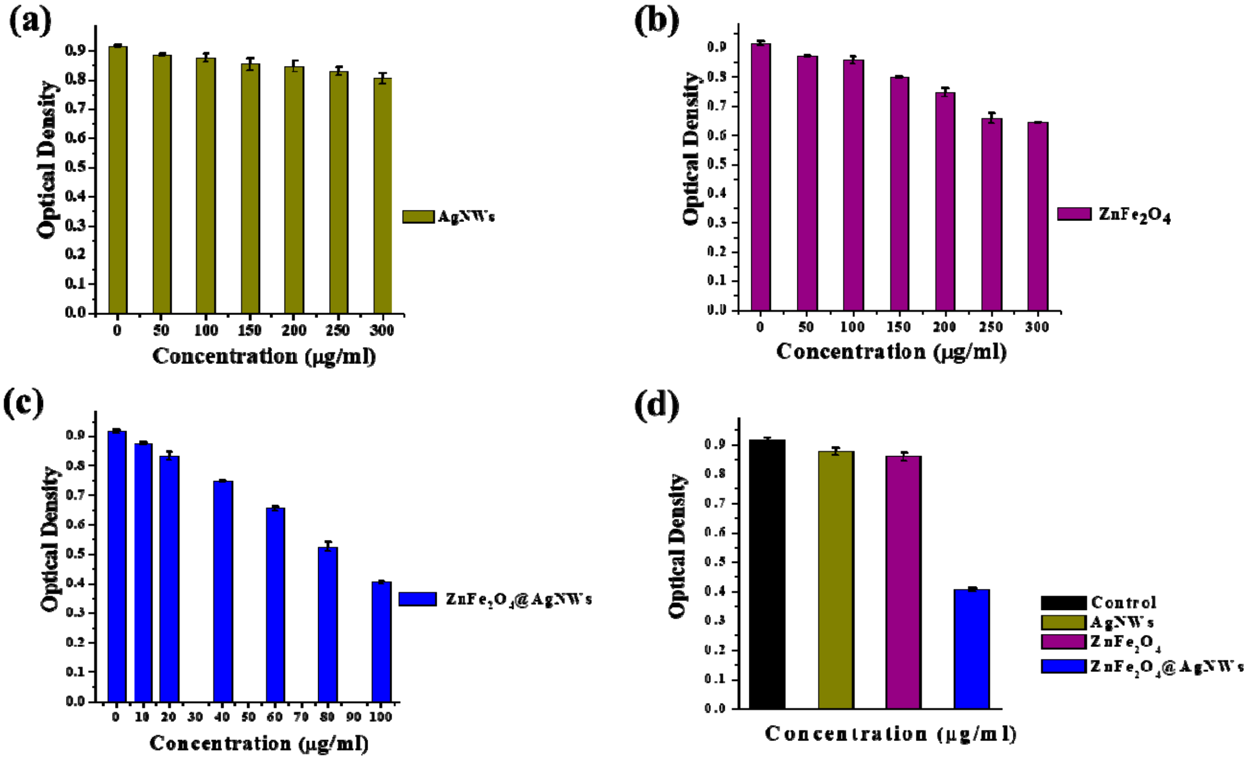

Figure S7. Optical density versus nanostructure concentration bar charts obtained from XTT cell proliferation assays: (a) AgNWs, (b) $ZnFe_2O_4$ NPs, (c) $ZnFe_2O_4$@AgNWs hybrid nanostructures, (d) comparative optical densities at a specific concentration of 100 µg/ml. The optical densities were converted from the light absorbance at the wavelength of 490 nm.

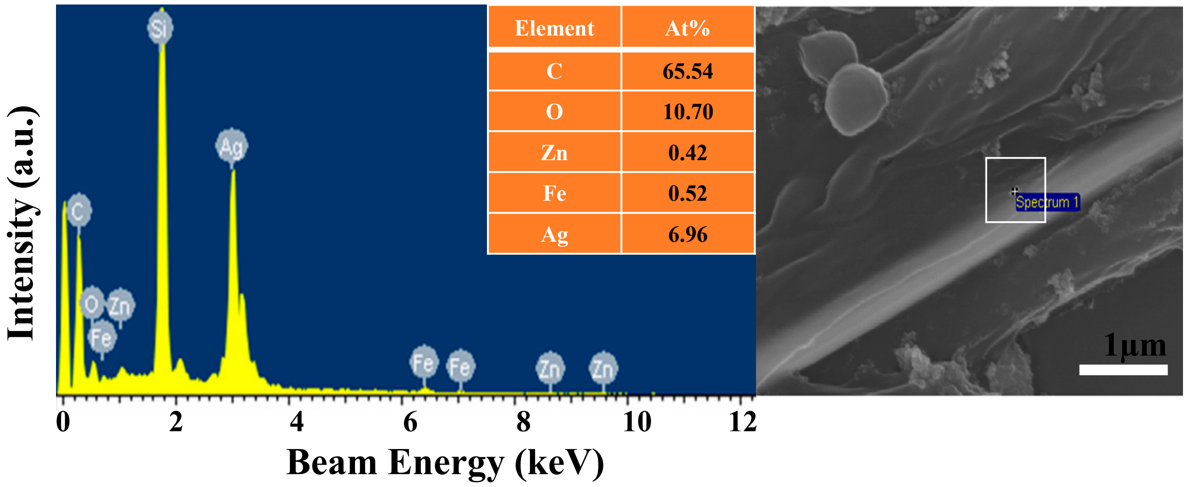

Figure S8. SEM-EDX spectrum showing the presence of every element in the biofilm sample treated with $ZnFe_2O_4$@AgNWs hybrid nanostructures (100 µg/ml). The intense silicon peak originated from the SiO_2 substrate used for sample preparation.

References

1 S. K. Shukla and T. S. Rao, *BioRxiv*, 2017, 100214, DOI: 10.1101/100214.

2 The Calorimetric reduction of XTT by cellular enzymes XTT cell proliferation assay kit instruction manual, https://www.atcc.org/~/media/Attachments/5/6/3/7/16747.ashx, 1988.

3 Molecular Probes Inc., LIVE/DEAD BacLight Bacterial Viability Kit Manual, https://tools.thermofisher.com/content/sfs/manuals/mp03224.pdf ,2001.
